# Врожденный изолированный гипогонадотропный гипогонадизм: клинический и молекулярно-генетический полиморфизм

**DOI:** 10.14341/probl12787

**Published:** 2021-08-06

**Authors:** К. Д. Кокорева, И. С. Чугунов, О. Б. Безлепкина

**Affiliations:** Национальный медицинский исследовательский центр эндокринологии; Национальный медицинский исследовательский центр эндокринологии; Национальный медицинский исследовательский центр эндокринологии

**Keywords:** синдром Кальмана, нормосмический гипогонадотропный гипогонадизм, молекулярно-генетический полимфорфизм, олигогенность

## Abstract

Врожденный изолированный гипогонадотропный гипогонадизм — группа заболеваний, вызванных нарушением секреции, действия гонадотропин-рилизинг-гормона (ГнРГ) и гонадотропинов. В половине случаев врожденный гипогонадотропный гипогонадизм сочетается с нарушением обоняния и носит название синдрома Кальмана. В настоящее время известно, что развитие данной группы заболеваний связано с мутациями в 40 генах, ответственных за функционирование гипоталамо-гипофизарно-гонадной оси. Гетерогенность клинической картины этой группы заболеваний, в том числе и в лечении, в значительной степени обуславливается различной молекулярно-генетической основой. Однако клинические проявления при одинаковых молекулярно-генетических дефектах могут варьировать даже в пределах одной семьи при одной и той же молекулярно-генетической основе заболевания. Корреляция между фенотипическими особенностями и генотипом обуславливает приоритетность поиска мутаций в определенных генах при сочетании гипогонадизма с врожденными пороками развития. В обзоре представлены данные о существенном вкладе в гетерогенность клинической картины заболевания олигогенного наследования. Кроме того, в работе поднимается вопрос о необходимости пересмотра современного определения и классификации врожденного изолированного гипогонадизма с учетом развития заболевания при мутациях в генах, не ассоциированных с закладкой и функционированием ГнРГ-секретирующих нейронов.

## Abstract (en)

Congenital isolated hypogonadotropic hypogonadism includes a group of diseases related to the defects of secretion and action of gonadotropin-releasing hormone (GNRH) and gonadotropins. In a half of cases congenital hypogonadism is associated with an impaired sense of smell. It’s named Kallmann syndrome. Now 40 genes are known to be associated with function of hypothalamus pituitary gland and gonads. Phenotypic features of hypogonadism and therapy effectiveness are related to different molecular defects. However clinical signs may vary even within the same family with the same molecular genetic defect. Genotype phenotype correlation in patients with congenital malformations prioritizes the search for mutations in candidate genes. There are data of significant contribution of oligogenicity into the phenotype of the disease are presented in the review. Moreover, an issue of current isolated hypogonadotropic hypogonadism definition and classification revision is raised in the review due to hypogonadotropic hypogonadism development while there are mutations in genes not associated with GNRH neurons secretion and function.

Врожденный изолированный гипогонадотропный гипогонадизм (ВИГГ) — группа заболеваний, обусловленных нарушением продукции гонадотропин-рилизинг-гормона (ГнРГ) и гонадотропинов, что проявляется задержкой полового развития и бесплодием. Развитие данного заболевания может быть обусловлено нарушением внутриутробной закладки нейронов, секретирующих ГнРГ, в ольфакторной плакоде (пластине), нарушением миграции этих нейронов вместе с ольфакторными нейронами в гипоталамус, нарушением секреции и/или действия самого ГнРГ, дефицитом гонадотропинов.Выделяют 2 основных фенотипа заболевания:

1. синдром Кальмана — сочетание гипогонадотропного гипогонадизма и гипосмии или аносмии, которое чаще наблюдается при нарушении закладки и миграции ГнРГ-секретирующих нейронов;

2. нормосмический вариант изолированного гипогонадотропного гипогонадизма может наблюдаться при нарушении секреции ГнРГ, мутациях в гене рецептора к ГнРГ, нарушении секреции гонадотропинов.

Несмотря на тот факт, что первые данные о мутациях в генах, ответственных за развитие гипогонадотропного гипогонадизма, были получены при изучении семейных случаев заболевания, в настоящее время большинство случаев гипогонадотропного гипогонадизма являются спорадическими [1]. Семейные случаи характеризуются неполной пенетрантностью и, соответственно, различной степенью выраженности клинических проявлений заболевания, от полной формы гипогонадизма до задержки пубертата. В настоящее время описаны формы гипогонадотропного гипогонадизма, которые наследуются по аутосомно-доминантному, аутосомно-рецессивному или Х-сцепленному типам.

Большинство случаев изолированного гипогонадотропного гипогонадизма описано у мужчин. Ранее считалось, что более низкая (до 10 раз) встречаемость данной патологии среди девочек (1:50 000–120 000) в сравнении с мальчиками (1:1000–30000) обусловлена Х-сцепленным типом наследования. Однако в настоящее время известно, что у большинства пациентов с данным синдромом выявлены формы гипогонадотропного гипогонадизма, наследуемые по аутосомно-доминантному и аутосомно-рецессивному типам [2]. Возможно, такие данные по гендерной распространенности обусловлены тем, что среди пациенток женского пола чаще встречаются неполные (парциальные) формы гипогонадизма [3], которые проявляются более мягким течением заболевания: может наблюдаться спонтанное развитие телархе и, редко, менархе, что затрудняет своевременную диагностику гипогонадизма [4]. Так, Shaw и соавт. в ретроспективном исследовании с участием 248 пациенток с гипогонадотропным гипогонадизмом показали, что у 51% женщин были развитые молочные железы, а 10% сообщили об 1–2 эпизодах менструальных кровотечений [4]. Вероятно, в детской практике парциальные случаи заболевания среди девушек-подростков остаются недиагностированными, чем обуславливается кажущаяся более высокая распространенность синдрома Кальмана и нормосмического варианта гипогонадотропного гипогонадизма среди юношей. Кроме того, в настоящее время описаны и другие генетические механизмы, приводящие к превалированию пациентов мужского пола при данном заболевании.

В ряде случае гипогонадотропный гипогонадизм сочетается с другими врожденными пороками. Так, например, при гипогонадизме вследствие мутаций в гене KAL1 в 40% случаев отмечаются неврологические нарушения в виде синкинезии рук, а гипогонадизм, обусловленный мутацией в гене FGFR1, может сочетаться с агенезией зубов и пороками развития кистей и стоп.

Гипогонадотропный гипогонадизм может входить в состав синдромальных патологий, например, CHARGE синдрома, синдрома Ваардербурга, септооптической дисплазии, синдрома Хартсфилда.

Таким образом, разнообразие фенотипа обуславливается молекулярно-генетическим полиморфизмом. Однако клинические проявления при одинаковых молекулярно-генетических дефектах могут варьировать даже в пределах одной семьи.

## МОЛЕКУЛЯРНО-ГЕНЕТИЧЕСКИЙ ПОЛИМОРФИЗМ КАК ОСНОВА ГИПОГОНАДОТРОПНОГО ГИПОГОНАДИЗМА

При врожденном гипогонадотропном гипогонадизме выявлены мутации в более чем 40 генах [5–8]. Однако установить молекулярно-генетическую основу заболевания удается только в 30–50% случаев, что, возможно, обуславливается различными методиками исследования [5].

Гены, связанные с развитием гипогонадотропного гипогонадизма, условно можно разделить на 3 группы: гены, ответственные за закладку ГнРГ-секретирующих нейронов в ольфакторной пластине и их миграцию в гипоталамус (FGF8, FGFR1, KAL1, PROK2, PROKR2, NELF и другие), за импульсную секрецию ГнРГ (GNRH1, GPR54, TAC3, TACR3 и др.) и ген, определяющий чувствительность рецепторов к ГнРГ (GNRHR) [4].

В таблице 1 представлены наиболее часто встречающиеся гены, мутации в которых ассоциированы с развитием гипогонадотропного гипогонадизма.

**Table:** Таблица 1. Наиболее часто встречающиеся гены, ассоциированные с синдромом Кальмана: частота встречаемости, тип наследования и клинические проявления

Ген	Частота встречаемостипри гипогонадизме	Ассоциированные клинические проявления	Ассоциированныесиндромы	Наследование
Ген аносминаKAL1 (ANOS1)	3,7–12% [14][16]	Бимануальная синкинезия. Агенезия почек	Не описано	X-сцепленное
Ген рецептора факторов роста фибробластов FGFR1	15–20% [5][32]	Пороки развития неба. Септооптическая дисплазия. Скелетные аномалии. Бимануальная синкинезия. Пороки развития кистей/стоп Пороки развития неба	Синдром Хартсфилда	АД
Гены факторов роста фибробластов FGF2*, FGF8*, FGF17*	Синдром Денди–Уокера
Ген рецептора ГнРГ (GNRHR)	6,6–11% [5][41]	Не описано специфических клинических проявлений	Синдром фертильного евнуха	АР
Ген хромодомена ДНК-зависимой хеликазы CHD7	6–16% [56][58]	Аплазия полукружного канала.Врожденная тугоухость.Аномалии сердца.Колобома	CHARGE-синдром	АД
Ген прокинетицина и его рецептора		Нарушения слуха, синкинезия рук, эпилепсия,нарушения сна, ожирение		АР
PROK2	3% [68]	
PROKR2*	6,6–7% [68]	Синдром утреннего сияния (патология диска зрительного нерва)

Мутации в генах, ответственных за развитие и миграцию ГнРГ-секретируюших нейронов из ольфакторной пластины в гипоталамус (FGF8, CHD7, HS6ST1, SOX10, SEMA3A,WDR11), ассоциированы с развитием гипогонадизма с аносмией, в то время как мутации в генах GNRHR, GNRH1, KISS1R, KISS1, TACR3, TAC3 чаще приводят к развитию нормосмического варианта гипогонадизма [8].

В настоящее время известно, что при одинаковых генетических дефектах степень нарушения обоняния у пациентов с гипогонадотропным гипогонадизмом может быть разной. Достоверно оценить наличие нарушений обонятельной функции у пациентов с синдром Кальмана возможно только методом ольфактометрии с помощью специальных наборов пахучих веществ. Ранее считалось, что обонятельные луковицы являются уникальным органом центральной нервной системы, для которого установлена корреляция между размером и функцией [1], однако Danda и соавт. в 2020 г. показали, что нарушение обонятельной функции не во всех случаях сопровождается изменениями обонятельных луковиц на МРТ головного мозга [8]: гипо- и аносмия могут наблюдаться у пациентов с интактными обонятельными луковицами. Причины развития аносмии или гипосмии при сохранных обонятельных луковицах у пациентов с синдромом Кальмана на данный момент неизвестны и требуют дальнейшего изучения.

По данным популяционных исследований установлено, что у пациентов с изолированным гипогонадотропном гипогонадизмом из различных этнических групп выявляются различные генетические нарушения. Bhagavath и соавт. в исследовании с участием более 300 пациентов с гипогонадотропным гипогонадизмом показали, что для северо-американской и турецкой популяций наиболее характерны мутации в гене рецептора ГнРГ (GNRHR) и в гене KAL1 [3]. По данным работы Guimaraes и соавт., для бразильской популяции наиболее характерны мутации в генах ANOS1 (он же KAL1), FGFR1 и GNRHR [5]. По данным исследования 2016 г. с участием 27 мальчиков с гипогонадизмом из Финляндии и 9 из Дании, наиболее типичными мутациями оказались нарушения в генах KAL1 и FGFR1 [9].

Несмотря на то что большинство случаев врожденного гипогонадотропного гипогонадизма являются спорадическими, очень важен детальный анализ родословной, так как доказано, что у половины пациентов с синдромом Кальмана имеются родственники с той или иной степенью нарушения репродуктивной функции. Рецессивный тип наследования можно предполагать в случае, если брак является близкородственным, или при наличии патологии у сибсов при фенотипически здоровых родителях. Однако большинство форм гипогонадотропного гипогонадизма наследуется по аутосомно-доминантному типу. Х-сцепленный тип наследования можно заподозрить в случае, если мальчик с гипогонадизмом и аносмией при фенотипически здоровой матери имеет родственника мужского пола со стороны матери, как правило, дядю [10], с конституциональной задержкой пубертата в анамнезе или гипогонадизмом.

## МУТАЦИИ В НАИБОЛЕЕ ЧАСТО ВСТРЕЧАЮЩИХСЯ ГЕНАХ: КОРРЕЛЯЦИЯ ГЕНОТИПА С ФЕНОТИПОМ

Ген аносмина KAL1

В 1991 г. был обнаружен первый ген, мутации в котором являются причиной развития синдрома Кальмана — KAL1 (Kallman syndrome 1, MIM 300836). Раньше этот ген называли ANOS1. Он состоит из 14 экзонов, а продуктом его экспрессии является белок аносмин-1, чем и объясняется предыдущее название. Аносмин является гликопротеином, состоящим из 680 аминокислот [11]. Он играет важную роль в формировании органов не только нервной, но и пищеварительной, опорно-двигательной и мочеполовой систем [12]. Аносмин влияет на процесс миграции ольфакторных и ГнРГ-секретирующих нейронов из назальной плакоды в гипоталамус во время внутриутробного развития, вследствие чего мутации в этом гене, как правило, ассоциированы с нарушением обоняния. Однако мутации в гене KAL1 могут приводить к гипогонадизму без гипо- и аносмии: так, по результатам исследования Sato et al. [13], среди 15 пациентов с мутациями в гене KAL1 у 2 пациентов не было выявлено нарушения обоняния.

По разным данным, распространенность мутаций в этом гене среди всех случаев ВИИГ составляет от 3,7% до 12% [14–16]. Среди генов, мутации в которых ассоциированы с развитием гипогонадизма, ген KAL1 является единственным, наследуемым Х-сцепленно [17], хотя ранее считалось, что он отвечает только за 70% Х-сцепленно передаваемых мутаций при гипогонадизме. Ген KAL1 расположен на коротком плече X-хромосомы (Xp22.3). Мутации в этом гене среди женщин с гипогонадизмом встречаются редко [18], что не позволяет рассматривать Х-сцепленное наследование как основную причину более высокой распространенности синдрома Кальмана среди мужчин: по результатам исследования 2019 г. с участием 39 женщин [5] наиболее часто выявляемыми мутациями оказались мутации в генах FGFR1 — 15%, GNRHR — 6,6% и PROKR2 — 6,6%, в то время как мутации в гене KAL1 выявлялись только в 6,2% случаев. Другое объяснение данного факта может быть в том, что гены, передающиеся Х-сцепленно и ответственные за правильную работу оси «гипоталамус-гипофиз-гонады», еще не открыты. К такому же выводу пришли финские ученые, которые обследовали 5 женщин с гипогонадотропным гипогонадизмом и у всех выявили мутации в гене FGFR1 [19]. Некоторые исследователи считают, что заболевание более часто встречается среди мужчин по причине достаточного образования аносмина у женщин и его взаимодействия с рецептором фактора роста фибробластов 1. Для гена KAL1 описан феномен избегания («Escape» phenomenon), при котором ген KAL1 у женщин при одной интактной Х-хромосоме «избегает» полной Х-инактивации. Таким образом, образуется достаточное количество аносмина для активации рецептора к фактору роста фибробластов, что может проявляться сохранной репродуктивной функцией при имеющемся парциальном дефекте гена у женщин [20–22].

Фенотип пациентов с мутациями в гене KAL1 вариабелен: у большинства развивается полная форма гипогонадизма [23], характеризующаяся объемом яичек менее 3 мл у мужчин и отсутствием развития молочных желез и менархе у женщин, однако встречаются неполные [24] и реверсивные формы [25][26]. Имеются данные о том, что пациенты с синдромом Кальмана и мутациями в гене KAL1 имеют вдвое меньший объем гонад в сравнении с пациентами с мутациями в генах NELF, CHD7, HS6ST1, FGF8/FGFR1, PROK2/PROKR2 [24]. Участие аносмина в формировании органов не только нервной, но и опорно-двигательной и мочеполовой систем объясняет частое сочетание синдрома Кальмана с аномалиями развития скелета, пороками развития средней линии, бимануальной синкинезией (зеркальные движения рук) и агенезией почек. Последняя встречается почти у каждого третьего пациента с синдромом Кальмана [16]. Бимануальная синкинезия долгое время считалась «уникальным» проявлением мутаций в гене KAL1, однако на данный момент известно, что синкинезия также может иметь место при мутациях в генах FGF8/FGFR1, PROK2/PROKR2, CHD7 и других, но при мутациях в гене KAL1 все же встречается в 4 раза чаще [24].

По данным исследования Costa-Barbosa и соавт. [24], среди мужчин с синдромом Кальмана и нарушениями в гене KAL1 ответ на пульсовую терапию ГнРГ был менее выражен в сравнении с группами пациентов с мутациями в генах FGF8/FGFR1, PROK2/PROKR2, CHD7 и с группой пациентов без установленной молекулярно-генетической причины заболевания: 50, 100, 100, 78% соответственно. Интересно, что среди женщин с синдромом Кальмана подобной тенденции не наблюдалось. Однако ограниченный размер выборки (34 пациента) не позволяет экстраполировать результаты исследования на всех пациентов с мутациями в гене KAL1.

В 1998 г. Maya-Nunez и соавт. описали клинический случай пациента с гипогонадотропным гипогонадизмом, аносмией и генерализованным ихтиозом. У данного пациента был диагностирован синдром «генных последовательностей» (contiguous gene syndrome), в основе которого — делеция 3 экзонов гена KAL1 и полная делеция гена стероидной сульфатазы STS [27], что привело к развитию ихтиоза. Данные гены расположены на Х-хромосоме (Xp22.3): ген KAL1 проксимальнее гена STS, а его 3’-конец обращен к теломере. Обнаружить микроделецию короткого участка Х-хромосомы с помощью метода секвенирования нового поколения, как правило, не удается [28]. Могут потребоваться полноэкзомное секвенирование [29] или проведение микроматричного анализа [30][31].

Гены факторов роста фибробластов FGF8/FGF2 и рецептора фактора роста фибробластов FGFR1

Мутации в гене рецептора факторов роста фибробластов 1-го типа FGFR1 (ранее KAL2) являются одними из самых часто встречающихся: мутация в гене FGFR1 выявляется у каждого пятого пациента с гипогонадотропным гипогонадизмом [32]. Ген рецептора фактора роста фибробластов расположен на коротком плече 8 хромосомы (8p11.23), состоит из 18 экзонов. Гипогонадотропный гипогонадизм при дефекте данного гена наследуется аутосомно-доминантно.

Продуктом экспрессии гена FGFR1 является рецептор фактора роста фибробластов 1-го типа — гликопротеин, содержащий 822 аминокислотных остатка. Взаимодействие рецептора фактора роста фибробластов с его эндогенными лигандами, факторами роста фибробластов, необходимо для запуска митоген-активируемого сигнального пути и играет важную роль в регуляции процессов эмбриональной дифференцировки и миграции ГнРГ-секретирующих и обонятельных нейронов [20].

Впервые нарушения закладки ГнРГ-секретирующих нейронов при дефектах гена FGFR1 были выявлены у генно-модифицированных мышей [33]. Ассоциация между мутациями в гене FGFR1 и синдромом Кальмана у человека была установлена в 2003 г., когда были описаны первые 4 семейных и 8 спорадических случаев заболевания [34].

Установлено, что мутации в гене FGFR1 могут выявляться у пациентов не только с синдромом Кальмана, но и с гипогонадизмом без нарушения обоняния. Фенотипические проявления, ассоциированные с инактивирующими мутациями в гене рецептора фактора роста фибробластов, варьируют от задержки пубертата, изолированной гипосмии до тяжелых форм гипогонадизма с множественными врожденными пороками развития [20][35].

Ген FGFR1 экспрессируется в различных клетках и тканях, чем обуславливается корреляция генотипа с фенотипом. Так, при сочетании гипогонадизма с пороками развития одной или обеих конечностей мутации в гене FGFR1 определяются в 90% случаев [36][37]. FGFR1 является первым геном-кандидатом, который рекомендуется исследовать у пациентов с пороками развития кистей и стоп [38]. Ранее считалось, что бимануальная синкинезия наблюдается только у пациентов с мутациями в гене KAL1, однако на данный момент известно, что синкинезия может наблюдаться и при дефектах в гене FGFR1 [39]. Кроме нарушений формирования конечностей, доказана корреляция между инактивирующими мутациями в генах факторов роста фибробластов (FGF8/FGF2) и аномалиями развития зубов, поэтому исследование этих генов является приоритетным при проведении генетического исследования у пациентов с гипогонадизмом и дентальной агенезией.

В 30% случаев у пациентов с мутацией в гене FGFR1 наблюдается расщелина губы и неба [8]. Пороки развития средней линии у данной когорты пациентов могут быть ассоциированы с голопрозэнцефалией и эктродактилией (отсутствие или недоразвитие одного или нескольких центральных пальцев кистей и стоп). Подобное сочетание врожденных пороков развития носит название синдрома Хартсфилда (Hartsfield syndrome) [40]. Степень выраженности клинических составляющих данного синдрома варьирует, что, по предположению некоторых исследователей, также объясняется высокой степень клинического полиморфизма при мутациях в данном гене [40].

В исследовании Li и соавт. 2020 г. с участием 14 мужчин с установленной мутацией в гене FGFR1 объем гонад был значимо ниже, чем в группе пациентов с гипогонадизмом без установленной генетической причины. Полиморфизм клинической картины проявляется и в ответе на заместительную терапию: доля пациентов, успешно ответивших на пульсовую терапию ГнРГ и гонадотропинами в виде индукции сперматогенеза, значимо не различалась в группах (76% vs 82%), в отличие от среднего времени до начала сперматогенеза: в группе пациентов с мутацией в гене FGFR1 добиться сперматогенеза удалось после 15 мес терапии, а в контрольной группе — после 10 мес (p<0,05), что подтверждает ранее выдвинутую гипотезу о том, что мутации в гене FGFR1 вызывают более тяжелые формы гипогонадизма [32].

Ген рецептора гонадотропин-рилизинг-гормона GNRHR

Мутации в гене рецептора ГнРГ (GNRHR, MIM 146110) встречаются у 7–11% пациентов с гипогонадотропным гипогонадизмом [41]. Ген расположен на длинном плече 4 хромосомы (4q21.2), состоит из 3 экзонов. Гипогонадотропный гипогонадизм при изменениях в этом гене наследуется по аутосомно-рецессивному типу.

Продуктом экспрессии гена является G-белок-ассоциированный рецептор, функционирующий на мембране гонадотрофов. Взаимодействие рецептора с ГнРГ приводит к активации различных внутриклеточных сигнальных путей, обеспечивающих регуляцию транскрипции генов гонадотропинов.

Впервые миссенс-мутации в гене GNRHR были описаны у брата и сестры с гипогонадотропным гипогонадизмом в 1997 г. [42]. Последующие клинические случаи также были описаны у сибсов [43][44]. Видимо, по этой причине считалось, что мутации в гене ГнРГ чаще встречаются при семейных формах заболевания, однако на данный момент известно, что около половины мутаций в гене ГнРГ являются спорадическими [45].

При мутациях в гене GNRHR, как правило, развивается гипогонадизм без нарушений обоняния [41].

Клинические проявления при мутациях в гене рецептора ГнРГ крайне вариабельны и зависят от степени инактивации рецептора: может развиваться как полный, так и парциальный гипогонадизм [46]. Beranova и соавт. установили, что у мужчин с мутациями в этом гене на момент установления диагноза объем яичек может быть как препубертатным, так и не отличаться от объема яичек у здоровых мужчин [45]. Кроме того, нарушения в гене ГнРГ могут приводить к развитию реверсивных форм гипогонадизма [47], которые, по данным исследования Gianetti и соавт., чаще наблюдаются при биаллельных мутациях, ассоциированных с парциальной инактивацией рецептора, в то время как моноаллельные мутации гена ГнРГ могут проявляться как тяжелыми формами гипогонадизма, так и не нарушать репродуктивную функцию вовсе [48].

Неполной степенью инактивации рецептора, по-видимому, объясняется определенная эффективность пульсовой терапии ГнРГ у данной когорты пациентов. Впервые Tamaya и соавт. В 2000 г. описали группу пациенток с гипогонадотропным гипогонадизмом вследствие мутаций в гене GNRHR, у которых отмечалась индукция овуляции в ответ на введение ГнРГ в пульсовом режиме [49]. Результаты исследований Seminara и соавт. и Abel и соавт. также доказывают, что резистентность рецептора у данной когорты пациенток может быть успешно преодолена [50][51]. Пульсовая терапия ГнРГ подтвердила свою эффективность и у мужчин с гипогонадизмом вследствие мутаций в гене GNRHR [52]. Предикторы эффективности пульсовой терапии у данной когорты пациентов не установлены, а ответ на терапию может различаться даже у сибсов. [53]

Ген хромодомена ДНК-зависимой хеликазы СHD7

Ген расположен на длинном плече 8 хромосомы (8q12). Состоит из 38 экзонов. Гипогонадизм, ассоциированный с мутацией в гене CHD7, наследуется аутосомно-доминантно. Продуктом экспрессии гена CHD7 является белок — хромодомен (CHRomatin Organisation MOdifier) АТФ-зависимой хеликазы. Как следует из названия, этот белок оказывает влияние на организацию и структуру хроматина. Продуктом экспрессии гена CHD7 является транскрипционный фактор — энхансер, влияющий на экспрессию других генов: есть данные о том, что CHD7 может регулировать экспрессию генов KAL1, FGFR1, PROK2 и PROKR2 [54]. Возможно, это одна из причин, по которой мутации в гене CHD7 могут приводить к развитию и синдрома Кальмана, и нормосмического гипогонадотропного гипогонадизма [55].

По результатам исследований частота мутаций в гене CHD7 у пациентов с врожденным гипогонадотропным гипогонадизмом составляет от 6 до 8% [56][57]. Однако результаты проведенного в 2019 г. исследования (Gonçalves и соавт.) с участием 50 пациентов с гипогонадотропным гипогонадизмом свидетельствуют о более высокой распространенности мутаций в гене CHD7 — до 16% [58], что, возможно, связано с объемом выборки.

Большинство мутаций в гене СHD7 являются спорадическими [57]. Анализируя семейные случаи заболевания, Pauli и соавт. пришли к выводу, что в 92% случаев мутации в гене СHD7 связаны с нарушениями в отцовском аллеле [59]. По данным исследования Marcos и соавт. [57], к гипогонадизму без нарушения обоняния и к синдрому Кальмана чаще приводят миссенс-мутации в гене СHD7, т.е. мутации, ассоциированные с образованием белка с большей молекулярной массой. При нонсенс-мутациях, мутациях сайтов сплайсинга и мутациях, приводящих к сдвигу рамки считывания, образуется более короткий белок с меньшей молекулярной массой, что чаще клинически проявляется СHARGE-синдромом [60].

## CHARGE-СИНДРОМ (OMIM 214800)

Это редкое тяжелое врожденное заболевание, распространенность которого составляет 1 на 8500–15 000 новорожденных. Интересно, что чаще СHARGE-синдром встречается среди девочек [17].

CHARGE-синдром впервые был описан в 1979 г. двумя работающими независимо друг от друга врачами — офтальмологом Хелен Хитнер (Helen Hitner) и морфологом Брайаном Холлом (Bryan Hall) [55]. Ученые описали когорту детей с множественными врожденными пороками развития и гипогонадизмом. Сначала CHARGE-синдром носил имена этих ученых (синдром Хитнер–Холла), пока в 1981 г. Pagon и соавт. не создали акроним СHARGE. Название синдрома является аббревиатурой, составленной из начальных букв его основных клинических проявлений, где «С» — coloboma — колобома, «H» — heart disease — пороки сердца, «A» — atresia of the choanae — атрезия хоан, «R» — retarded growth and development — задержка роста и развития, «G» — genital hypoplasia — гипоплазия наружных половых органов и «E» — ear anomalies — пороки развития органов слуха, которые могут проявляться тугоухостью. Кроме этого, у пациентов с CHARGE-cиндромом могут наблюдаться расщелина неба, пороки развития пищевода, почек и гипопитуитаризм. В 1988 г. Blake и соавт. [55, 61] систематизировали клинические проявления при CHARGE-синдроме и выделили большие и малые критерии заболевания [62], а в 2009 г. критерии были пересмотрены и из больших критериев были исключены поражения черепных нервов. В 2016 г. были предложены новые диагностические критерии, в которых центральное место отводится мутациям в гене CHD7 [63]. Такое предложение кажется обоснованным с учетом высокой частоты встречаемости атипичных и парциальных форм синдрома [61][63][64]: так, например, гипогонадотропный гипогонадизм встречается в половине случаев, а большинство мутаций в гене CHD7 ассоциировано только с нарушениями слуха и пороками развития средней линии. Однако выявить мутации в гене СHD7 у пациентов с CHARGE-синдромом удается лишь в 58–90% случаев [61]. По результатам исследования Lalani и соавт. с участием 110 пациентов, среди тех, у кого диагноз был подтвержден результатами молекулярно-генетического исследования, в сравнении с пациентами без установленной мутации значимо чаще наблюдались пороки развития сердца, колобома и асимметрия лица, обусловленная односторонним парезом лицевого нерва [61].

## Ген прокинетицина и его рецептора PROKR2, PROK2

Ген PROKR2 расположен на коротком плече 20-й хромосомы (20p12.3). Продуктом гена PROKR2 является рецептор прокинетицина 2-го типа. Взаимодействие рецептора с лигандом, прокинетицином, продуктом гена PROK2 (3p13), играет важную роль в нейрогенезе обонятельной луковицы [65]. Гипогонадотропный гипогонадизм при дефектах данных генов наследуется аутосомно-доминантно.

Впервые мутации в этих генах у пациентов с синдромом Кальмана были описаны в 2006 г. Dode и соавт. [65] диагностировали 10 различных мутаций в гене PROKR2 и 4 мутации в гене PROK2. У одного из пациентов, кроме мутации в гене PROKR2, была выявлена мутация в гене KAL1.

До 2008 г. считалось, что прокинетициновый сигнальный путь оказывает свое влияние только на формирование обонятельных луковиц и, соответственно, может наблюдаться только у пациентов с гипогонадизмом и нарушением обоняния. Однако в 2008 г. Cole и соавт. [66] выявили мутации в гене PROKR2 у 4 пациентов с гипогонадизмом без нарушения обоняния. Отсутствие мутаций в других ассоциированных с гипогонадизмом генах позволили Сole и соавт. предположить, что развитие нормосмического гипогонадотропного гипогонадизма у этих пациентов не обусловлено олигогенным наследованием. Еще ранее, в 2007 г. Pitteloud и соавт. установили, что мутации в гене самого прокинетицина 2-го типа тоже могут приводить к развитию гипогонадизма без нарушения обоняния [67].Распространенность мутаций в генах прокинетицина и его рецептора исследована только среди пациентов с синдромом Кальмана и составляет 7% для мутаций в гене PROKR2 и 3% для мутаций в гене PROK2 [68].

У пациентов с мутациями в генах PROK2/PROKR2, кроме гипогонадизма, могут иметь место нарушения слуха, синкинезия рук, эпилепсия, нарушения сна, ожирение [67][69]. Ожирение и нарушения сна, вероятно, объясняются влиянием прокинетицина на циркадные ритмы сна и бодрствования и пищевое поведение [68][69].

Для пациентов с моноаллельными мутациями в генах PROKR2 и PROK2 характерны более мягкие формы гипогонадизма, чем для пациентов с биаллельными мутациями: пациенты с моноаллельными мутациями имеют больший объем яичек, более высокие базальные уровни тестостерона, лютеинизирующего (ЛГ) и фолликулостимулирующего гормонов (ФСГ) ( [69]. Высокие уровни ЛГ у некоторых пациентов связывают с «двойным» влиянием мутаций в гене PROKR2 на репродуктивную систему и развитием первичной резистентности гонад — данный ген экспрессируется в сперматогенном эпителии и интерстиции, что может проявляться первичным гипогонадизмом [66, 70].

## МУТАЦИИ В ГЕНАХ, НЕ АССОЦИИРОВАННЫХ С ЗАКЛАДКОЙ И ФУНКЦИОНИРОВАНИЕМ ГНРГ-СЕКРЕТИРУЮЩИХ НЕЙРОНОВ

За развитие изолированного гипогонадотропного гипогонадизма могут отвечать мутации в генах, не ассоциированных с формированием, миграцией и работой ГнРГ-секретирующих нейронов. С 1990-х гг. описывались единичные клинические случаи мутаций β-субъединиц ЛГ и ФСГ, сопровождающихся развитием гипогонадизма. Однако один и тот же полиморфизм может проявляться как гипогонадотропным гипогонадизмом, так и гипергонадотропным гипогонадизмом или не приводить к снижению репродуктивной функции вовсе. Так, результаты исследования с участием более 2500 тысяч мужчин подтвердили связь между полиморфизмом в гене LHB, кодирующем бета-субъединицу ЛГ (так называемый V-LHB полиморфизм, Trp8Arg/Ile15Thr), и сниженным уровнем ЛГ. При этом, по результатам работы Punab и соавт. 2015 г. с участием более 1500 мужчин, тот же самый полиморфизм проявлялся повышением уровня ЛГ, то есть формально приводил к гипергонадотропному гипогонадизму, но не влиял на показатели спермограммы [71]. Данные этого исследования частично согласуются с результатами исследования с участием 120 женщин, в котором данный полиморфизм также был ассоциирован с развитием гипергонадотропного гипогонадизма, однако проявлялся снижением репродуктивной функции [72].

Спектр клинических проявлений, ассоциированных с мутациями в бета-субъединице ФСГ, также широк: мутации в этом гене приводят к развитию как полных, так и парциальных форм гипогонадизма [73][74].

Описано несколько клинических случаев успешного лечения препаратами хорионического гонадотропина человека (ХГЧ) пациентов с гипогонадотропным гипогонадизмом, вследствие мутации в гене LHB: лечение препаратами ХГЧ в течение 6 мес в дозе 4000–5000 МЕ в неделю у 28-летнего пациента привело к нормальным показателям спермограммы [75]. Другой клинический случай лечения 30-летнего мужчины с мутацией в бета-субъединице ЛГ препаратами ХГЧ также позволил добиться не только андрогенизации, но и индукции сперматогенеза [76]. Исследований, подтверждающих высокую эффективность терапии ХГЧ, на больших группах не проводилось, что, по-видимому, связано с очень низкой встречаемостью данного генетического дефекта. Лечение пациентов с мутациями в гене, кодирующем бета-субъединицу ФСГ, вероятно, также приводит к хорошим результатам. Однако данное предположение основывается только на единичных описаниях успешного применения препаратов ФСГ у пациенток с первичной аменореей [77][78]. В исследовании Matthews и соавт. пациентка на фоне терапии смогла в естественном цикле зачать ребенка [79]. Уже на 5-й день применения препаратов ФСГ наблюдались повышение уровня эстрадиола, ингибина B, а также развитие множественных фолликулов [80]. В статье Kottler и соавт. также приводится описание клинического случая девушки с мутацией в гене FSHB, которая получала лечение рекомбинантным ФСГ в течение 10 дней, на фоне чего наблюдалось повышение уровня ингибина B и эстрадиола [81].

Кроме мутаций в генах, ответственных за функционирование ГНРГ-секретирующих нейронов, известно, что к гипогонадотропному гипогонадизму также могут привести инактивирующие мутации в генах лептина (LEP) и его рецептора (LEPR) [82]. Лептин оказывает влияние на секрецию гипоталамическими нейронами нейропептида Y, кисспептина и проопиомеланокортина, которые, в свою очередь, регулируют пульсовую секрецию ГнРГ [83]. Экзогенное введение лептина пациенту с дефицитом лептина позволило инициировать собственное половое развитие [84].

Описанные случаи выходят за рамки устоявшихся представлений о природе изолированного врожденного гипогонадотропного гипогонадизма как заболевания, ассоциированного с мутациями в генах, ответственных за правильную работу ГнРГ-секретирующих нейронов. Случаи развития гипогонадизма при мутациях в генах, кодирующих бета-субъединицы ФСГ и ЛГ, а также в генах лептина и его рецептора, свидетельствуют о необходимости пересмотра современного определения и классификации заболевания [74].

## ОЛИГОГЕННАЯ ПРИРОДА НАСЛЕДОВАНИЯ

В 1990 г. английский врач L.J. Hipkin и соавт. описали случай развития синдрома Кальмана у одного близнеца из пары, в то время как его однояйцевый близнец наблюдался только с гипосмией, но вовремя вступил в пубертат и имел нормальный уровень тестостерона [85]. У родителей и сестры близнецов не наблюдалось нарушений обоняния и репродуктивной функции. Еще раньше, в 1985 г., детскими эндокринологами из ФРГ был описан случай развития гипогонадизма с аносмией у одной девочки из пары монозиготных близнецов, в то время как ее сестра-близнец не имела задержки полового развития, но наблюдалась с аносмией [86]. Такой клинический полиморфизм может быть обусловлен неполной пенетрантностью или влиянием эпигенетических факторов, но нельзя исключить и потенциальный вклад олигогенности, так как Jonsson и соавт. в 2021 г. было доказано, что геном монозиготных близнецов не идентичен [87]: по данным исследования Raivio. и соавт., мутации в гене рецептора фактора роста фибробластов FGFR1 проявлялись различными нарушениями репродуктивной функции, от неполных форм до реверсивного течения гипогонадизма [88], что, вероятно, обуславливается выявленными мутациями и в других генах (GNRHR, PROKR2 и FGF8). По данным исследования Sykiotis и соавт. также указывается, что дигенные нарушения чаще выявляются именно у пациентов с мутациями в гене FGFR1 [52].

Известно, что 80% случаев гипогонадотропного гипогонадизма обусловлены мутацией в одном гене, 12% — мутациями в двух (дигенные нарушения) и 2,5% — мутациями в более чем двух генах (олигогенное наследование) [17]. В европейском консенсусе 2015 г. по врожденному гипогонадотропному гипогонадизму приведены данные о том, что олигогенный характер могут носить до 20% случаев заболевания [82]. Однако только олигогенной природой наследования объяснить фенотипическую гетерогенность заболевания не представляется возможным.

## НЕПОЛНЫЕ И РЕВЕРСИВНЫЕ ФОРМЫ ГИПОГОНАДИЗМА

Неполные, или парциальные, формы гипогонадизма диагностируются у пациентов с объемом гонад >4 мл перед началом лечения. По данным исследования Hao и соавт. с участием 122 мужчин с врожденным гипогонадотропным гипогонадизмом, с развитием неполных форм чаще ассоциированы мутации в генах FGFR1, PROKR2 и CHD7. В исследовании также сравнивались результаты лечения гонадотропинами у пациентов с парциальными и полными формами заболевания: после двух лет терапии индукция сперматогенеза была отмечена у 92% пациентов с парциальным гипогонадизмом и у 75% с полными формами заболевания [89].

От 10 до 20% случаев гипогонадизма характеризуется реверсивностью, то есть, по сути, является поздним пубертатом [82]: наличие реверсивных форм доказано для мутаций в генах GNRHR, PROKR2, CHD7 [6] и KAL1 [90]. Так, описан клинический случай пациента с двумя миссенс-мутациями в 11 экзоне гена KAL1, который получал терапию хорионическим гонадотропином человека. Через 1,5 года от начала приема мужчина самостоятельно прекратил терапию ХГЧ, а через 3 мес после прекращения терапии ХГЧ, несмотря на сниженный объем эякулята, снижение концентрации, подвижности и количества сперматозоидов, пациентом в естественном цикле без применения вспомогательных репродуктивных технологий был зачат ребенок без фенотипических проявлений, ассоциированных с гипогонадотропным гипогонадизмом [25]. Таким образом, диагностирована реверсивная форма заболевания. Возможно, прекращение терапии ХГЧ способствовало «растормаживанию» оси гипоталамус-гипофиз-гонады. Одна из мутаций, диагностированных у данного пациента, а именно мутация в кодоне 514 11 экзона, приводящая к замене аминокислоты глутамина на лизин, была уже ранее описана у 6 пациентов с синдромом Кальмана [2]. Следовательно, именно сочетание 2 миссенс-мутаций привело к реверсивному, более мягкому течению, заболевания. Через 4 мес с момента зачатия ребенка у пациента появились жалобы на эректильную дисфункцию, в связи с чем потребовалось назначение терапии тестостероном. Реверсивные формы гипогонадизма не всегда сопровождаются долговременным или пожизненным сохранением репродуктивной функции и часто требуют назначения заместительной гормональной терапии [82].

Предикторов развития реверсивной формы на данный момент не выявлено, а результаты молекулярно-генетического исследования не во всех случаях могут помочь клиницистам в дифференциальной диагностике заболевания, особенно с учетом вклада олигогенного наследования в фенотипический полиморфизм.

## ЗАКЛЮЧЕНИЕ

Врожденный гипогонадотропный гипогонадизм характеризуется генетическим и клиническим полиморфизмом. Однако различные фенотипические проявления заболевания, от задержки пубертата до полных форм гипогонадизма, могут наблюдаться даже в одной семье при одном и том же молекулярно-генетическом дефекте. На данный момент накоплено достаточно данных, чтобы утверждать, что такая неполная пенетрантность клинических проявлений может быть обусловлена олигогенностью.

Корреляция между определенными фенотипическими проявлениями и генотипом может помочь клиницисту не только в выборе приоритетных для молекулярно-генетического исследования генов, но и подходящего метода исследования.

Данные молекулярно-генетического исследования могут стать основой персонализированной терапии. Есть основания полагать, что наиболее эффективна пульсовая терапия ГнРГ у пациентов с мутациями в гене рецептора ГнРГ за счет различной степени его инактивации.
